# Supplementary material for: Efficacy and Safety of Kudzu Flower–Mandarin Peel on Hot Flashes and Bone Markers in Women during the Menopausal Transition: A Randomized Controlled Trial
Source: Nutrients. 2020 Oct 22;12(11):3237. doi: 10.3390/nu12113237 (PMC7690627; doi:10.3390/nu12113237)
Supplement: Supplementary file 1 [file nutrients-12-03237-s001.pdf]

Supplementary Table 1. Safety parameters

Variables	Placebo (*n* = 42)	KM (*n* = 42)	Estimate	*p*-value
**Vital signs**				
SBP (mmHg): below 120 mmHg				
Week 0	118.2 ± 1.6	118.2 ± 2.1	-3.82	0.12
Week 12	120.8 ± 2.2	115.6 ± 2.4		
p-value	0.199	0.358		
DBP (mmHg): below 80 mmHg				
Week 0	73.2 ± 1.4	72.7 ± 1.6	-1.77	0.363
Week 12	75.1 ± 1.4	72.0 ± 1.9		
p-value	0.189	0.971		
Pulse rate (beats/min): 60~100 beats/min				
Week 0	76.0 ± 1.4	75.7 ± 1.8	-1.68	0.428
Week 12	77.0 ± 1.9	76.5 ± 1.9		
p-value	0.226	0.907		
Body temperature (℃): 36.1~37.2 ℃				
Week 0	36.2 ± 0.1	36.3 ± 0.1	-0.09	0.481
Week 12	36.1 ± 0.0	36.2 ± 0.1		
p-value	0.73	0.174		
**Hematologic analysis**				
White blood cell (10^3/μL): 3.4~9.6 x 10^3/μL				
Week 0	5.8 ± 0.2	5.2 ± 0.2	0.34	0.361
Week 12	5.4 ± 0.2	5.2 ± 0.2		
p-value	0.118	0.753		
Red blood cell (10^6/μL): 3.9~5.1 x 10^6/μL				
Week 0	4.3 ± 0.1	4.3 ± 0.0	-0.04	0.415
Week 12	4.3 ± 0.0	4.3 ± 0.0		
p-value	0.613	0.516		
Hemoglobin (g/dL): 11.6~15 x g/dL				
Week 0	13.0 ± 0.1	13.2 ± 0.2	0.04	0.775
Week 12	13.0 ± 0.1	13.1 ± 0.2		
p-value	0.816	0.521		
Hematocrit (%): 35.5~44.9 %				
Week 0	39.4 ± 0.4	40.0 ± 0.4	-0.01	0.987
Week 12	39.9 ± 0.4	40.0 ± 0.5		
p-value	0.165	0.158		
Platelet (10^3/μL): 157~371 x 10^3/μL				
Week 0	272.6 ± 9.6	255.3 ± 10.4	8.4	0.314
Week 12	277.1 ± 9.2	268.4 ± 10.4		
p-value	0.595	0.047		
Neutrophil (%): 40~60 %				
Week 0	53.7 ± 1.3	53.5 ± 1.3	1.06	0.547
Week 12	51.8 ± 1.0	53.2 ± 1.1		
p-value	0.15	0.524		
Lymphocyte (%): 20~40 %				
Week 0	36.1 ± 1.2	36.8 ± 1.3	0.32	0.529
Week 12	37.8 ± 0.9	36.2 ± 1.1		
p-value	0.345	0.064		
Monocyte (%): 2~8 %				
Week 0	7.2 ± 0.2	7.0 ± 0.3	0.05	0.371

Week 12	7.1 ± 0.3	7.2 ± 0.2		
p-value	0.342	0.026		
Eosinophil (%): 1~4 %				
Week 0	2.2 ± 0.3	2.0 ± 0.2	-1.56	0.304
Week 12	2.5 ± 0.4	2.6 ± 0.4		
p-value	0.17	0.957		
Basophil (%): 0~3 %				
Week 0	0.76 ± 0.05	0.71 ± 0.04	0.08	0.787
Week 12	0.79 ± 0.06	0.78 ± 0.05		
p-value	0.696	0.994		
**Blood biochemical analysis**				
AST (U/L): 7~55 U/L				
Week 0	24.0 ± 1.7	23.3 ± 2.0	1.67	0.358
Week 12	21.4 ± 1.1	21.3 ± 0.9		
p-value	0.012	0.186		
ALT (U/L): 8~45 U/L				
Week 0	22.8 ± 2.8	21.8 ± 3.8	2.64	0.352
Week 12	17.8 ± 1.5	17.4 ± 1.3		
p-value	0.004	0.106		
ALP (U/L): 40~129 U/L				
Week 0	73.1 ± 3.1	64.1 ± 2.3	2.45	0.248
Week 12	74.6 ± 1.5	68.5 ± 3.4		
p-value	0.413	0.014		
BUN (mg/dL): 7~20 mg/dL				
Week 0	13.1 ± 0.6	13.6 ± 0.5	-0.32	0.672
Week 12	13.0 ± 0.5	13.5 ± 0.6		
p-value	0.786	0.377		
Creatinine (mg/L): 5.9~10.4 mg/L				
Week 0	6.8 ± 0.2	7.2 ± 0.1	-0.02	0.116
Week 12	6.8 ± 0.2	6.9 ± 0.1		
p-value	0.643	0.007		
eGFR ($mL/min/1.73m^2$): over 90 $mL/min/1.73m^2$				
Week 0	93.0 ± 2.4	86.5 ± 1.7	4.06	0.08
Week 12	93.2 ± 2.5	90.8 ± 2.1		
p-value	0.72	0.005		

Values are presented as mean ± SE. Estimates and *p*-values were obtained from a linear mixed effect model adjusted for covariates. KM, an extract mixture of kudzu flower and mandarin peel; SBP, systolic blood pressure; DBP, diastolic blood pressure; AST, aspartate aminotransferase; ALT, alanine transaminase; ALP, alkaline phosphatase; BUN, blood urea nitrogen; eGFR, estimated glomerular filtration rate.
